# The impact of timing of in utero marijuana exposure on fetal growth

**DOI:** 10.3389/fped.2023.1103749

**Published:** 2023-05-16

**Authors:** Phoebe Dodge, Katherine Nadolski, Haley Kopkau, Victoria Zablocki, Kaya Forrestal, Beth A. Bailey

**Affiliations:** Central Michigan University College of Medicine, Mount Pleasant, MI, United States

**Keywords:** fetal growth, development, prenatal marijuana/cannabis exposure, pregnancy smoking, cannabinoids

## Abstract

**Objective:**

To examine whether timing of *in utero* marijuana exposure independently and negatively impacts fetal growth, and if these effects are global or specific to certain growth parameters.

**Study design:**

The two study groups were marijuana users (*N* = 109) and a randomly selected control group of biochemically verified non-users (*n* = 171). Study data were obtained *via* manual abstraction of electronic medical records.

**Results:**

After control for significant confounders, regression results indicated significant (*p* < .05) decrease in newborn weight following first trimester marijuana exposure only (−154 g) and following marijuana exposure throughout gestation (−185 g) compared to controls. There were also significant deficits in head circumference following marijuana exposure in the first and second trimester only (−.83 cm) and marijuana exposure throughout pregnancy (−.79 cm) compared to controls. Newborn length was not significantly predicted by marijuana exposure.

**Conclusions:**

Timing of marijuana exposure appears to play a key role in specific fetal growth deficits, with exposure throughout gestation most detrimental. However even first trimester exposure may result in decreased weight. Timing and amount of use could be confounded in this study as those who quit early in pregnancy may have been lighter users than those who continued throughout pregnancy. More research is clearly needed to better understand the role of amount and timing of *in utero* marijuana exposure in predicting different aspects of fetal growth, however, this study suggests that women should be encouraged to avoid marijuana use at any point in pregnancy.

## 1. Introduction

*Cannabis sativa* (marijuana) is the most popular illicit drug in the United States and is most prevalent among users aged 18–25 years old (1). To date, twenty-one states plus Washington D.C have legalized recreational marijuana use in the past decade. With legalization has come increased perception of safety, and research has revealed that many dispensaries have recommended marijuana to pregnant women to ease symptoms of pregnancy, especially morning sickness (2). Indeed, a recent study found that women in states with legalized recreational marijuana were more likely to consume marijuana during the preconception, prenatal, and postpartum periods vs. women in states where marijuana is illegal (3). Another study noted that 18.1% of pregnant women met criteria for marijuana abuse and/or dependence (4). The American College of Obstetricians and Gynecologists advise that “obstetrician–gynecologists should be discouraged from prescribing or suggesting the use of marijuana for medicinal purposes during the period before pregnancy, and during pregnancy and lactation” (5).

Although it is widely known in the medical community to discourage the use of marijuana during pregnancy, the exact effects of marijuana on the developing fetus are unclear. Previous studies have found *in utero* marijuana exposure in general to impact developmental outcomes, including gestation length, health status at birth, body length, birth weight, and head circumference, and neurodevelopment (6–9). However, many studies reported no significant findings after control for confounding (10–13). This is an important consideration as marijuana users differ from non-users in many ways, including substantially higher rates of tobacco use which is known to negatively impact fetal growth. Due to the legalization and commercial growing of marijuana, there is evidence that today's marijuana is more potent and is consumed in higher quantities than it was even a decade ago, leading to increased urgency for definitive findings (14).

In addition to the consideration of confounding, few studies have investigated the influence that timing of marijuana use during gestation could have on the results (7–9). Marijuana use in pregnancy has been found to lead to a compromise in the transfer of oxygen and nutrients to the fetus *via* impaired development of fetal-placental circulation including altered maternal blood space, decreased fetal capillary area, and greater collagen deposition (15), suggesting a possible mechanism for impacts on fetal growth, especially later in pregnancy. An international study showed that continued use of cannabis at 15 weeks of pregnancy was associated with significantly lower birthweight, head circumference, birth length, and gestational age at birth, along with more frequent severe neonatal morbidity or death, suggesting that even early gestational exposure, and especially continued use, may be detrimental (16). The goal of this study was to expand upon recent research and investigate whether the timing of *in utero* marijuana exposure predicted fetal growth, and to examine how various growth parameters may be differentially impacted while carefully controlling for potential confounders. We expected that marijuana use throughout pregnancy may have a greater impact on fetal growth than marijuana use earlier in pregnancy. Due to previous findings on the various risks associated with small birth size (17–21), our findings have the potential to inform future clinical practice and guidelines surrounding marijuana use in pregnancy to improve clinical outcomes.

## 2. Methods

### 2.1. Participants and procedure

Study participants were drawn from women receiving prenatal care at an academic obstetric practice that included patients from both the immediate urban area and the larger surrounding rural region. The study was approved by the Institutional Review Board of the affiliated hospital. Patients were initially eligible for study inclusion if they had a singleton pregnancy, were at least 17 years of age, and delivered during the five-year study window (2016–2020). Patients meeting these criteria were identified *via* an automated search of the electronic medical records, which resulted in a list of 1,738 women who entered prenatal care during the study period. The medical records of these women were then manually reviewed for information related to pregnancy substance use. As part of a larger study, women with different types of substance use were classified into exposure groups, along with a randomly selected group of control women. Additionally eliminated from the sample were women for whom no biologic assessment of substance use was available, and women with documented pregnancy alcohol or hard illicit drug use or prescription drug misuse. This resulted in a sample of 519 women in the parent study.

As the current study focused on prenatal marijuana exposure, of relevance to this investigation were the Marijuana Only exposure group, and the Control Group. Participants were classified into the Marijuana Only group if they self-reported marijuana use or had at least one laboratory test positive for marijuana. Laboratory testing was ordered as clinically indicated and for most women in the sample included at least one urine drug screen at entry to prenatal care and another urine drug screen at delivery. The majority of the sample had additional urine drug screens performed during pregnancy (82%), had a urine drug screen performed on their newborn after delivery (90%), or had newborn cord blood testing results available (78%). Other than a positive indicator for marijuana, women in the Marijuana Only group had no additional indications for substance use: they denied tobacco, electronic cigarette, alcohol, and all other illicit drug use during pregnancy, and all laboratory testing was negative for all substances other than marijuana. Additionally, study participants did not have any prescriptions for opioids, benzodiazepines, or barbiturates during pregnancy. The Control Group met al.l these criteria as well, in addition to having no positive indicator for marijuana use. A total of 109 women met the criteria and were classified as Marijuana Only. A total of 171 women met the criteria and were classified into the Control Group.

Following group assignment, electronic medical records were manually reviewed, with all study data abstracted onto study data collection sheets. Abstractions were performed by three of the medical student study investigators, with regular team discussions and oversight by the senior investigator to ensure data collection fidelity. Study data were entered into an electronic spreadsheet by the study coordinator, assisted by a trained undergraduate student and the medical student study investigators, with frequent checks for entry accuracy.

### 2.2. Variables

The primary predictor was drug exposure status, grouped as Marijuana Only or Control as described above. Of particular interest was timing of marijuana exposure, with the Marijuana Only group further subdivided into those who used marijuana only prior to 14 weeks' gestation, those who used marijuana only prior to 26 weeks' gestation, and those who continued to use marijuana after that point, based on self-report and urine drug screen results at different points during gestation. Primary outcomes were three birth size parameters: weight in grams, length in centimeters, and head circumference in centimeters.

Additional data were collected to construct variables to describe the study sample and control for confounding. These included several maternal background factors: age, race/ethnicity (coded as White non-Hispanic, African American, Hispanic, and Other), marital status (coded as married vs. single), highest level of education (collected as number of years and analyzed as high school graduate or less vs. any level of post-secondary education), and medical insurance (coded as Medicaid/none vs. private). Maternal medical factors collected included parity, pre-pregnancy BMI (calculated and analyzed as a continuous variable), pregnancy weight gain in pounds, gestational age in weeks/days at first prenatal medical visit, diagnosis of existing or gestational diabetes, diagnosis of hypertension (chronic or pregnancy induced), gestational age at birth in weeks/days, and infant sex.

### 2.3. Data analysis

Because the study sample was intended to include all eligible marijuana users, *a priori* power analysis was not conducted. However, following completion of data collection, a power analysis was performed to ensure that adequate power was available to answer the primary research question. Based on the current sample size of 109 marijuana exposed newborns and 171 controls and using established means and standard deviations for U.S. populations, power was 95% to detect a 10% difference in birth weight, and 88% to detect an 8% difference.

Chi-square analyses and t-tests were used to examine the association between group membership (Marijuana Only vs. Control) and maternal background and medical factors. Differences on the three fetal growth parameters between Control participants, those who used Marijuana Only prior to 16 weeks, and those who continued to use Marijuana Only after 16 weeks on the three continuous birth outcomes, were examined using separate linear regression analyses. All background factors significantly associated with group membership at *p* < .10 plus gestational age at delivery were entered stepwise on the initial steps, followed by entry of timing of marijuana exposure (no exposure was the reference group) on the final step. All data were managed, and all analyses were performed using IBM SPSS version 26.

## 3. Results

Background characteristics of the study participants by marijuana use status are presented in Table 1. Participants who used marijuana were significantly younger, more likely to be single, less likely to have an education beyond high school, and more likely to be covered by Medicaid compared to control women. Additionally, women who used marijuana gained significantly more weight during pregnancy compared to non-users.

**Table 1:** Participant characteristics by marijuana exposure status.

	Unexposed Controls (*n* = 171)	Marijuana Exposed (*n* = 109)	t/*χ*^2^	*p*
Mother's age (years)	25.4 (4.8)	22.7 (3.6)	5.06	<.001
Mother's race (% white non-Hispanic)	41.5%	34.9%	2.87	.163
Mother's marital status (% single)	67.1%	91.7%	22.55	<.001
Mother's highest level of education (% H.S. graduate or less)	47.4%	72.6%	13.43	<.001
Mother's medical insurance (% Medicaid or none)	68.4%	88.1%	14.12	<.001
Parity	1.1 (1.2)	1.1 (1.2)	.15	.883
Pre-pregnancy BMI	28.4 (7.1)	28.6 (7.3)	.16	.875
Pregnancy weight gain (lb)	23.1 (12.0)	28.9 (15.1)	3.53	<.001
Gestational age at first prenatal visit	14.6 (4.0)	14.3 (3.9)	.69	.492
Gestational diabetes (%)	11.1%	5.5%	2.57	.109
Pregnancy induced hypertension (%)	9.9%	5.6%	1.68	.194
Gestational age at delivery (wk)	38.6 (2.2)	38.9 (2.5)	1.16	.248
Gender (% male)	53.8%	44.0%	.16	.690

Table 2 shows the regression results of birth outcomes based on timing of marijuana exposure during pregnancy. After controlling for significant confounders, analyses indicated significant (*p* < .05) differences in newborn weight for women who used marijuana during the first trimester only and women who used marijuana throughout gestation compared to non-marijuana users. There were also statistically significant head circumference deficits for those who used marijuana in the first and second trimester only and for women who used marijuana throughout pregnancy compared to non-users. Newborn length was not significantly predicted by marijuana use at any time point in pregnancy.

**Table 2:** Regression results predicting birth size from timing of marijuana exposure.

Outcome predictors	Adjusted mean difference ± standard error	t	*p*
Weight (gm)			
Marijuana 1st trimester only	−154.0 +/− 75.4	2.04	.043
Marijuana 1st and 2nd trimester only	−164.1 +/− 104.3	1.57	.117
Marijuana throughout gestation	−185.1 +/− 68.1	2.72	.007
Length (cm)			
Marijuana 1st trimester only	−.27 +/−.45	.60	.557
Marijuana 1st and 2nd trimester only	−.61 +/−.38	1.61	.109
Marijuana throughout gestation	−.94 +/−.57	1.65	.101
Head circumference (cm)			
Marijuana 1st trimester only	−.47 +/−.27	1.73	.086
Marijuana 1st and 2nd trimester only	−.83 +/−.41	2.01	.046
Marijuana throughout gestation	−.79 +/−.32	2.47	.014

All potentially significant factors (*p* < .10) in Table 1, were considered as confounders in regression analyses, in addition to gestational age at delivery. Analyses were performed with entry of gestational age at delivery on the first step, stepwise entry for all potential confounders on the next steps, followed by entry of timing marijuana exposure (no exposure was the reference group) on the final step. Results presented are from the final step with all significant factors included.

## 4. Discussion

The results of this study support the hypothesis that marijuana use throughout pregnancy, compared to use only early in pregnancy, has the greatest impact on fetal growth, with both newborn weight and head circumference impacted. However, size parameters may be differentially impacted as weight appears to be reduced somewhat by marijuana exposure even if it only occurs early in gestation, while reduced head circumference was predicted only when exposure continued through the second trimester. Most fetal weight gain and growth occurs in the third trimester with an estimated fetal weight velocity accelerating throughout pregnancy and peaking at 35 weeks of gestation (22). This may be a potential reason for larger size deficits linked to late pregnancy, compared to early pregnancy, exposure. Birth length was not predicted by marijuana exposure in this study, but a potentially clinically meaningful deficit of nearly a centimeter resulted following exposure throughout gestation, a finding that approached but did not reach statistical significance. Thus, findings suggest a timing effect, with growth deficits generally greatest for those exposed to marijuana throughout gestation, but with meaningful impacts from even early gestational exposure depending on the growth parameter examined.

Our findings are consistent with previous research that found significant differences in birth weight and head circumference, but not birth length, based on marijuana use in pregnancy (7–9). Additionally, our findings about the role of timing of exposure may explain some of the inconsistencies across published studies that did not account for when during pregnancy marijuana exposure occurred. And while exposure beyond the second trimester may be the key for the largest growth effects, we cannot rule out the possibility that timing of exposure may have been a proxy for both duration (as late use was confounded with duration of use), and amount of exposure given we were not able to reliably determine amount of exposure in our study. It could be that those who failed to quit early in pregnancy were the ones consuming greater amounts of or more potent marijuana. And we certainly know they used marijuana over a longer period of time, increasing total exposure.

Low birth weight and decreased head circumference are associated with neurological and psychological issues, health complications in childhood, and the development of various non-communicable diseases in adulthood (17–19). Multiple studies suggest an increased risk of cognitive delay and neurodevelopmental disorders (including ADHD) among babies born at a low birth weight (17, 20, 21). Low birth weight has also been associated with obesity later in adolescence or adulthood with the possibility of early catch-up growth as a contributing factor to this (18). Low birth weight with early catch-up growth is also suggested to increase the risk of childhood hypertension (18). In the last decade, a large study aimed to assess whether low birth weight, small head circumference and low Apgar scores were predictors for developmental abnormalities throughout childhood and into adulthood (19). The study provided evidence that small head circumference was a significant predictor of psychological functioning, including depression, social dysfunction, and somatic symptoms. The same study also found that low birth weight was associated with a 2-fold increased risk for depression and social dysfunction while other studies have suggested that there is an association between low birth weight and adult psychiatric disorders (19, 21). The effects described in these studies have followed size deficits of the magnitude or even smaller than those found in the current study. Given the identified association with decreased size, then, it is important for both healthcare providers and patients to be aware of the potential implications of continuing to use marijuana during pregnancy. Our findings suggest that the earlier in pregnancy that marijuana use is discontinued the better chance a fetus has for normal growth and ultimately better child health and development.

Our study had several strengths. We were able to expand upon previous research by including timing of marijuana use during pregnancy when examining specific fetal outcomes. Additionally, given the known high rates of denial of substance use during pregnancy, we required self-report and biochemical confirmation of no use for our control group. We also eliminated alcohol and other drug use, including tobacco use, as possible factors for differences between groups.

One limitation of this study was the relatively limited sample size, which was a function of both our single site study and participant characteristics that led to study exclusion, especially polysubstance use among participants and limited routine biological testing. A further limitation was the possibility of missing a drug exposure following patient denial and the limited detection window for urine drug screens. Additionally, there is always the potential for human error, both on the provider end during charting and the researcher end during retrospective chart review and data input. Accuracy was ensured by multiple individuals throughout the process to minimize these errors. Finally, while data were derived from a large academic obstetric practice in the Midwestern U.S., generalizability of the findings is limited without further involvement of more heterogeneous participants.

While this study established significant findings of marijuana's effects on certain growth parameters based on timing of use, we did not have a method to compare the total amount of substance being consumed. Thus there was likely variability in regularity of use and the potency or amount of intake with each use among the study participants. These findings would be difficult to establish using retrospective chart review of routine obstetric visits. However, future prospective studies could produce more specific reports of drug use from participants to explore how total amount and even potency of marijuana use may impact fetal growth parameters.

In conclusion, marijuana use throughout gestation predicted significant deficits in birth weight and head circumference at delivery. Marijuana use during the first trimester resulted in significant deficits in newborn weight, with downward trends that were not statistically significant for other growth parameters. Marijuana use throughout the second trimester also had downward trends for all parameters, with a statistically significant impact on head circumference. While there were no statistically significant effects on birth length, downward trends related to ongoing marijuana exposure may suggest findings that are meaningful clinically. Healthcare providers should be aware of these implications of marijuana use in pregnancy, taking notice of the greater effects due to use later in gestation. It is important to encourage patients to stop using marijuana as soon as they become pregnant to reduce fetal growth risks and potential long term adverse health and developmental outcomes in offspring. These findings may also be used to influence commercial practices at cannabis dispensaries where marijuana use during pregnancy has not always been discouraged (2). Overall research into this topic can benefit from future studies with broader patient populations that further prospectively investigate the impact of amount, timing, and potency of marijuana consumption during pregnancy.

## Data Availability

The datasets presented in this article are not readily available because the datasets generated and analyzed for this study are the property of CMU Medical Education Partners and Covenant Health System, not the study authors. As such, data are not publicly available, and any request for access would need to be made to and approved by both CMU Medical Education Partners and Covenant Health System. Requests to access the datasets should be directed to beth.bailey@cmich.edu.
